# Associations between diffusion kurtosis imaging metrics and neurodevelopmental outcomes in neonates with low-grade germinal matrix and intraventricular hemorrhage

**DOI:** 10.1038/s41598-024-67517-5

**Published:** 2024-07-16

**Authors:** Chunxiang Zhang, Meiying Cheng, Zitao Zhu, Kaiyu Wang, Brianna F. Moon, Sheng Shen, Bohao Zhang, Zihe Wang, Lin Lu, Honglei Shang, Chi Qin, Jinze Yang, Yu Lu, Xiaoan Zhang, Xin Zhao

**Affiliations:** 1 Harvard Medical School, Boston, MA USA; 2 Department of Radiology, The Third Affiliated Hospital of Zhengzhou University, Zhengzhou, China; 3 Henan International Joint Laboratory of Neuroimaging, Zhengzhou University, Zhengzhou, China; 4 Wuhan University, Wuhan, China; 5 GE Healthcare, MR Research China, Beijing, China; 6 Zhengzhou University, Zhengzhou, China

**Keywords:** Neonates, Germinal matrix hemorrhage-intraventricular hemorrhage, Diffusion kurtosis imaging, Neurodevelopmental outcomes, Quantitative MRI, Neonatal brain damage, White matter injury

## Abstract

Diffusion Kurtosis Imaging (DKI)-derived metrics are recognized as indicators of maturation in neonates with low-grade germinal matrix and intraventricular hemorrhage (GMH-IVH). However, it is not yet known if these factors are associated with neurodevelopmental outcomes. The objective of this study was to acquire DKI-derived metrics in neonates with low-grade GMH-IVH, and to demonstrate their association with later neurodevelopmental outcomes. In this prospective study, neonates with low-grade GMH-IVH and control neonates were recruited, and DKI were performed between January 2020 and March 2021. These neonates underwent the Bayley Scales of Infant Development test at 18 months of age. Mean kurtosis (MK), radial kurtosis (RK) and gray matter values were measured. Spearman correlation analyses were conducted for the measured values and neurodevelopmental outcome scores. Forty controls (18 males, average gestational age (GA) 30 weeks ± 1.3, corrected GA at MRI scan 38 weeks ± 1) and thirty neonates with low-grade GMH-IVH (13 males, average GA 30 weeks ± 1.5, corrected GA at MRI scan 38 weeks ± 1). Neonates with low-grade GMH-IVH exhibited lower MK and RK values in the PLIC and the thalamus (P < 0.05). The MK value in the thalamus was associated with Mental Development Index (MDI) (r = 0.810, 95% CI 0.695–0.13; P < 0.001) and Psychomotor Development Index (PDI) (r = 0.852, 95% CI 0.722–0.912; P < 0.001) scores. RK value in the caudate nucleus significantly and positively correlated with MDI (r = 0.496, 95% CI 0.657–0.933; P < 0.001) and PDI (r = 0.545, 95% CI 0.712–0.942; P < 0.001) scores. The area under the curve (AUC) were used to assess diagnostic performance of MK and RK in thalamus (AUC = 0.866, 0.787) and caudate nucleus (AUC = 0.833, 0.671) for predicting neurodevelopmental outcomes. As quantitative neuroimaging markers, MK in thalamus and RK in caudate nucleus may help predict neurodevelopmental outcomes in neonates with low-grade GMH-IVH.

## Introduction

Germinal Matrix Hemorrhage-Intraventricular Hemorrhage (GMH-IVH) is a prevalent intracranial complication in preterm infants, despite advancements in their care^1^. Neonates with GMH-IVH continue to pose a significant concern in this vulnerable population. Approximately 25 to 50% of cases involve clinically silent GMH-IVH, detected through routine cranial ultrasound screening^2^. According to the classification of Volpe^3^, the term "low-grade" GMH-IVH encompasses Grade I (bleeding confined to the germinal matrix or GMH plus IVH occupying < 10% of the lateral ventricular area.) or Grade II (GMH plus IVH occupying 10 to 50% of the lateral ventricular area). These neonates are at risk of neurodevelopmental impairment^4^. However, such impairment often takes over a year to manifest in those with low-grade GMH-IVH. Recognizing the benefits of early intervention, including rehabilitative treatment for improved prognosis, researchers are actively seeking early biomarkers in these neonates. MRI has gained prominence among imaging tools for marking these biomarkers, given its noninvasive nature and ability to provide qualitative and quantitative insights into the brains of neonates with low-grade GMH-IVH.

Neonates experiencing low-grade GMH-IVH may exhibit subtle white matter microstructural abnormalities that often go undetected with conventional cranial ultrasonography and routine MRI. Addressing this challenge, diffusion kurtosis imaging (DKI) emerges as a valuable MRI tool capable of quantifying these microstructural abnormalities^5^. Among the DKI metrics, mean kurtosis (MK) and radial kurtosis (RK) stand out, offering direct physical relevance to the diffusion tensor^6^. MK reflects average diffusion kurtosis, while RK represents kurtosis along the radial direction, providing insight into the microstructural complexity of imaged tissue^7^. While conventional diffusion tensor imaging (DTI) sequence has shown potential in detecting mild GMH-IVH, they are limited by their reliance on a Gaussian diffusion model, unable to address the challenge of cross-fiber orientation in brain tissue^8^. In contrast, DKI, rooted in the non-Gaussian diffusion of water in biological systems, presents a promising technology. Its applications extend to various conditions, including prematurity^9^, spinal cord imaging^10^, and acute bilirubin encephalopathy^11^. Associations with neurodevelopmental outcomes have been explored, revealing adverse motor and language abilities in preterm infants with severe intraventricular hemorrhage^12^. Notably, these findings suggest a potential relationship between regional DKI-derived metrics and neurodevelopmental outcomes in preterm infants with severe intraventricular hemorrhage, yet few studies have specifically evaluated the correlation between DKI-derived metrics and neurodevelopmental outcomes in neonates with low-grade GMH-IVH.

This study aimed to investigate whether early DKI metrics in neonates with low-grade GMH-IVH were associated with subsequent neurodevelopmental outcomes. To test this hypothesis, we prospectively enrolled neonates, measuring regional fractional anisotropy (FA), MK, and RK derived from DKI. Subsequently, we correlated these values with neurodevelopmental outcomes assessed by the Bayley Scales of Infant Development at 18 months of age.

## Materials and methods

### Participants

This is a retrospective study conducted at our institution, and written informed consent was obtained from each patient's guardian. The study design received approval from the Ethics Committee of the Third Affiliated Hospital of Zhengzhou University, and the protocol of the project conformed to the ethical standards of the Helsinki Declaration of 1975, revised in 2008. All data were anonymized before processing the MRI data.

Preterm neonates admitted to our hospital between January 2020 and March 2021 were enrolled. The inclusion criteria for the case group were (a) neonates diagnosed with low-grade GMH-IVH through transcranial ultrasound (Supplemental 1), (b) gestational age (GA) less than 34 weeks, (c) birth weight less than 1500 g, and (d) completion of the BSID-III scale assessment at 18 months. The inclusion criteria for the control group were (a) GA less than 34 weeks, (c) birth weight less than 1500 g, and (d) completion of the BSID-III scale assessment at 18 months. Exclusion criteria for both case and control groups included neonates with (a) congenital malformation, infection, or metabolic diseases, (b) brain lesions other than low-grade GMH-IVH, (c) any structural abnormality on prior ultrasounds, (d) incomplete DKI imaging, and (e) inadequate coverage of the whole brain or motion artifacts on the MRI. These excluded conditions have been linked to poor neurodevelopmental outcomes^13^ and may obscure or confound our evaluation of the significance of quantified MRI values for outcome prediction. Initially, there were a total of 70 neonates recruited (low-grade GMH-IVH group (n = 30) and control group (n = 40)) that met the inclusion and exclusion criteria.

### MRI data acquisition

Neonates were scanned at a corrected GA of 38 weeks ± 1 using a 3 T MRI scanner (Pioneer, GE Healthcare, Milwaukee, WI) (total n = 70), equipped with a dedicated 8-channel phased-array head coil. To minimize movement, neonates were positioned on an immobilization pillow. Mini muffs (Natus Medical Inc., San Carlos, CA) were utilized to reduce noise from the MRI scanner. Throughout the procedure, all neonates were monitored using pulse oximetry and electrocardiographic monitoring. Detailed MRI acquisition protocols are listed in Table S1.

An advanced diffusion imaging technique, DKI, corrects Gaussian model defects and quantifies the deviation of each voxel from free diffusion. DKI sequence parameters included TR = 2000 ms, TE = 103 ms, employing 30 diffusion directions for b-values of 0, 1000, and 2000 mm^2^/s, respectively. Slice thickness was set at 4.0 mm without a gap, the field of view at 256 mm × 256 mm, and the acquisition time at 7.5 min.

### MRI data analysis

The regular MRI data were transmitted to the hospital's PACS for incidental findings screening. Three radiologists, with 30, 10, and 5 years of experience in neuroradiology, respectively, and blinded to clinical data apart from basic participant information displayed on PACS, independently reviewed each scan soon after its completion. This review aimed to ensure the absence of clinically diagnosable incidental findings, and no variability measurement was conducted for the screening process.

All MR imaging data preprocessing and analysis were performed using the FMRIB Software Library (FSL, www.fmrib.ox.ac.uk/fsl). Skull stripping was performed using the brain extraction tool (BET) function. Eddy current and head motion artifact were corrected in FSL by aligning diffusion-weighted images to the first b0 image with an affine DKI transformation with 12 degrees of freedom. The three commonly used DKI measures, including FA, MK, and RK were calculated using standard methods^14^. Additionally, we utilized ITK-SNAP 4.0 (http://www.itksnap.org/pmwiki/pmwiki.php) for region of interest (ROI) delineation^15^. ITK-SNAP offers semi-automatic segmentation via active contour methods, manual delineation, and image navigation, along with various supporting utilities. ROIs were drawn for the thalamus, caudate nucleus, posterior limbs of the internal capsule (PLIC), genu of the corpus callosum (GCC), and occipital lobe. Because these ROIs all have been related to the long-term outcome^3,16–18^. We found that DTI measurement comparison shows that there is no statistical difference between the two hemispheres of the brain, so the average value of both sides is taken as the result of ROI. Each regional value was obtained for both right and left hemispheres, and their mean values were utilized for the final analysis^19^. Two neuroradiologists, with 12 and 15 years of experience, independently measured all ROIs on T_1_WI. They reached a consensus on the image outline before measurement, and the final data used for analysis represented the average of their measurements. Subsequently, a third neuroradiologist, possessing 15 years of experience, examined the results.

### Neurodevelopmental outcomes at 18 months

Neurodevelopmental outcomes were evaluated in neonates at 18 months of corrected age using the 2nd edition of the Bayley Scales of Infant and Toddler Development. A trained examiner conducted the examination, assessing the Mental Development Index (MDI) and the Psychomotor Development Index (PDI). The MDI measures cognition, evaluating environmental responsiveness, sensory and perceptual abilities, learning, memory, and early language and communication skills. The PDI assesses motor skills, covering both gross and fine motor skills. The Bayley Scales of Infant and Toddler Development is a widely used test, with higher scores indicating better outcomes. Additional details are available in S2.

### Statistical analysis

To compare clinical characteristics and MRI findings between the two groups classified according to the neurodevelopmental outcome, we conducted the *t* test, Mann–Whitney U test, or χ^2^ analysis. Group differences in DKI measures were adjusted for confounding parameters (sex, gestational age and weight) by using a general linear regression model^20^. Due to the large number of ROIs and MRI metrics in this study, we employed Storey False Discovery Rate (FDR) correction for multiple comparisons. The FDR *P* Value < 0.1 was deemed statistically significant. Additionally, Spearman correlations were utilized to assess the relationship between quantitative MRI metrics and neurodevelopmental outcome scores. Receiver operating characteristic (ROC) analysis was performed to recognize the diagnostic performance of DKI metrics in different brain regions for predicting adverse neurodevelopmental. Intraclass correlation coefficients (ICC) were computed to analyze interobserver agreement; further details are available in Table S2. SPSS software, version 28.0 (IBM SPSS Statistics), was utilized for the analysis. A *p* value < 0.05 was deemed statistically significant.

## Results

### Participants

**Figure 1:**
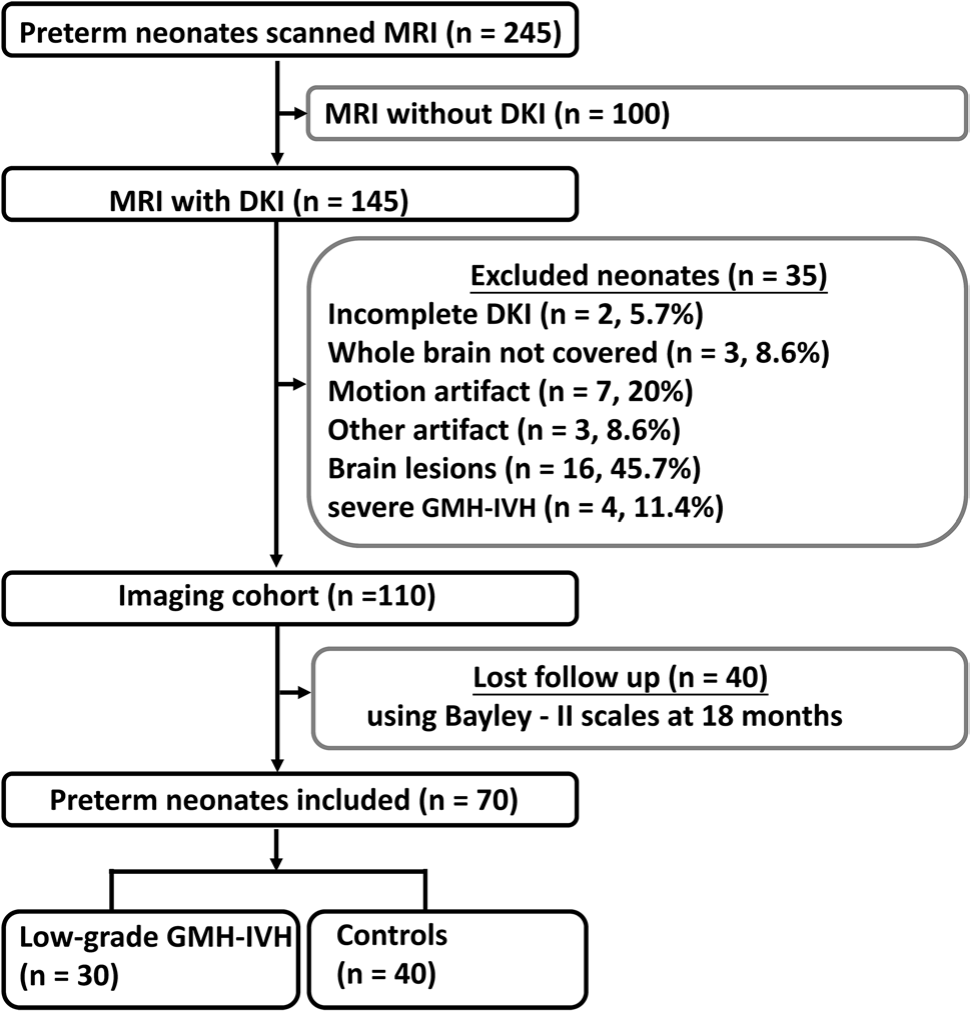
Flowchart shows inclusion and exclusion criteria for neonates. *DKI* Diffusion kurtosis imaging, *Bayley—II* Bayley Scales of Infant and Toddler Development 2nd Edition, *low-grade GMH-IVH* low-grade Germinal Matrix Hemorrhage-Intraventricular Hemorrhage.

A retrospective evaluation was conducted on brain MRI studies of 245 neonates. DKI and synthetic MRI sequences were performed in 145 of these neonates. Exclusions were made for 35 neonates (24.1%): 16 had other brain lesions (including 13 punctate WM lesions, two arteriovenous malformations, and one callosal dysgenesis), 4 had severe GMH-IVH, 2 had incomplete DKI, 3 had brain areas not covered by the scans, and 10 had DKI sequences affected by motion artifacts or other artifacts. Consequently, the MRI studies of 110 out of 145 (75.9%) neonates were included in the imaging cohort. Forty neonates with lost follow-up using Bayley—II Scales were excluded. The study ultimately included 70 neonates, with 30 cases showing the presence of low-grade GMH-IVH (illustrated in Fig. 1). Among these, 40 (57.1%, 40/70) were controls (17 females, average GA 30 weeks ± 1.3, corrected GA at MRI scan 38 weeks ± 1), while the remaining 30 (42.9%, 30/70) were neonates with low-grade GMH-IVH (22 females, average GA 30 weeks ± 1.5, corrected GA at MRI scan 38 weeks ± 1). Detailed demographic information for the neonates and their mothers is presented in Table 1. Analysis of this matched cohort revealed no differences in perinatal, maternal, and other factors.

**Table 1:** Demographic and Baseline Neonate Characteristics.

Characteristic	Neonates with low-grade GMH-IVH (n = 30)	Preterm neonates (n = 40)	*P* value*
Neonatal characteristics			
Gestational age at birth (wk)	30 ± 1.3	30 ± 1.5	0.14
Birth weight (g)	1240 ± 192	1297 ± 138	0.28
Birth head circumference (cm)	31 ± 1	31 ± 1	0.98
Birth length	35 ± 3	35 ± 4	0.38
Apgar score at 1 min	4 ± 0.4	4 ± 0.8	0.35
Apgar score at 5 min	6 ± 0.4	6 ± 0.6	0.34
Corrected gestational age at MRI scan (wk)	38 ± 1	38 ± 1	0.92
Hydrocortisone treatment (n)	10 ± 1	12 ± 2	0.52
Days of intubation (days)	16 ± 2	14 ± 3	0.72
Sex			0.67
Female	17 (56)	22 (55)	…
Male	13 (44)	18 (45)	…
Retinopathy of prematurity	4 (13)	7 (17)	0.56
Patent ductus arteriosus	13 (43)	17(42)	0.77
Necrotizing enterocolitis	1 (3)	3 (7.5)	0.52
Chronic liver disease	1 (3)	1 (2)	0.85
Infections	3 (13)	6 (15)	0.71
Major surgery	1 (3)	2 (5)	0.79
Chorioamnionitis	5 (16)	6 (15)	0.82
Maternal characteristics			
Maternal age at birth (y)	24 ± 4	25 ± 5	0.65
Method of delivery			0.13
Vaginal	10 (33)	13 (33)	…
Cesarean	20 (67)	27 (67)	…
Risk level of pregnancy			0.06
High risk	4 (14)	10 (25)	…
Not high risk	26 (86)	30 (75)	…
Maternal level of education			0.26
Primary/secondary	22 (73)	29 (72)	…
Under/postgraduate	8 (27)	11 (28)	…
Breastfeeding at all	25 (83)	34 (85)	0.13
Smoking during pregnancy	1 ± 1	3 ± 1	0.57
Alcohol consumption during pregnancy	2 ± 2	3 ± 1	0.46

Unless otherwise indicated, data are presented as mean ± standard deviation for continuous variables and as number (percentage) for categorical variables. Low-grade GMH-IVH = low-grade GMH-IVH = low-grade Germinal Matrix Hemorrhage-Intraventricular Hemorrhage.

*Fisher exact test for categorical variables and t test for continuous variables.

### MRI characteristics

**Figure 2:**
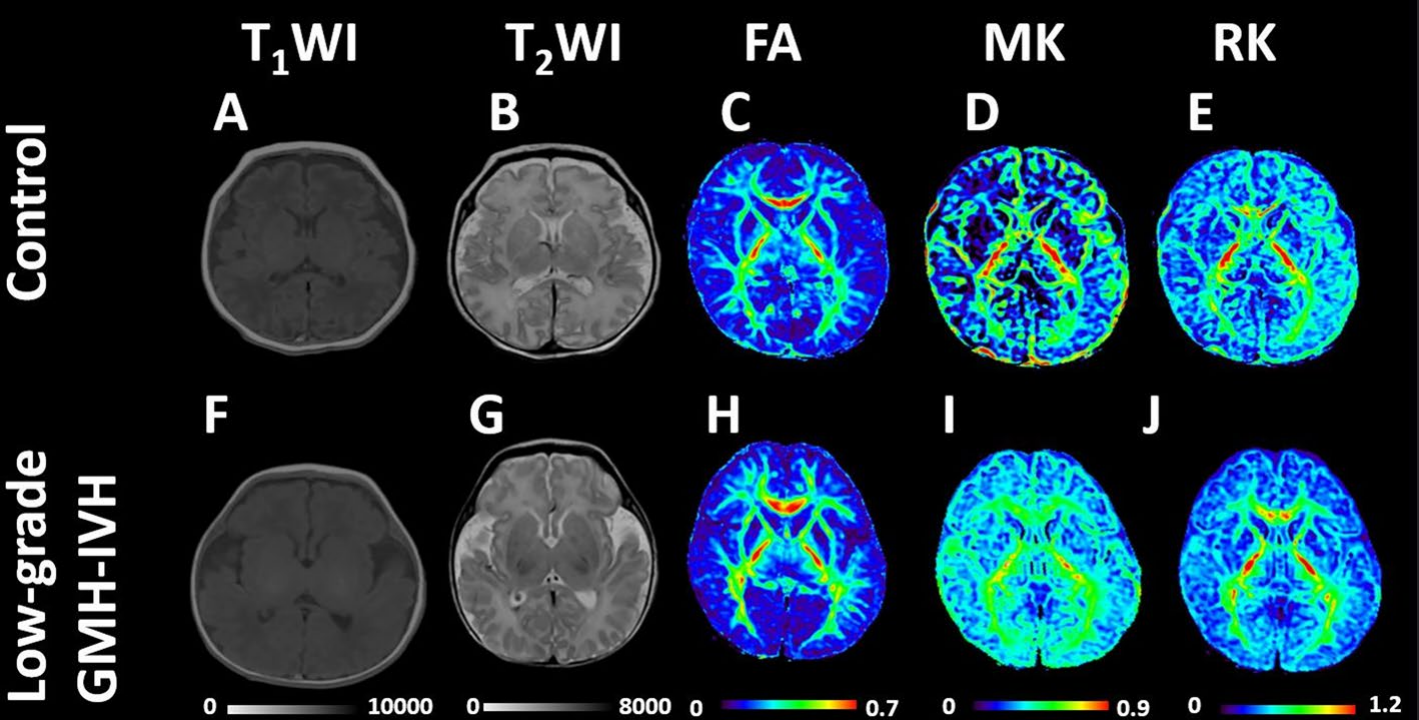
Representative neonatal images (T_1_WI, T_2_WI, FA, MK, and RK) without (top row) and with (bottom row) low-grade GMH-IVH. (A-E) showcases a male neonate with a gestational age of 31 weeks, corrected gestational age at MRI scan is 38 weeks, with Bayley—II scores of MDI and PDI at 125 and 121. (F-J) features a male neonate with a gestational age of 30 weeks, corrected gestational age at MRI scan is 38 weeks, and Bayley—II scores of MDI and PDI at 93 and 100. *DKI* Diffusion kurtosis imaging, *FA* Fractional anisotropy, *MK* mean kurtosis, *RK* radial kurtosis, *low-grade GMH-IVH* low-grade Germinal Matrix Hemorrhage-Intraventricular Hemorrhage, *Bayley—II* Bayley Scales of Infant and Toddler Development 2nd Edition, *MDI* Mental Development Index, *PDI* Psychomotor Development Index.

Representative examples from two distinct neurodevelopmental outcome groups underwent DKI scans. An infant without encephalopathy is illustrated in Fig. 2A–E. This male neonate, born at 31 weeks with a weight of 1640 g, had a corrected gestational age of 38 weeks at the MRI scan. The Bayley-II scores for MDI and PDI were 125 and 121, respectively. Another neonate with low-grade GMH-IVH is depicted in Fig. 2F–J. This male neonate, born at 30 weeks with a weight of 1693 g, had a corrected gestational age of 38 weeks at the MRI scan. The Bayley-II scores for MDI and PDI were 93 and 100, respectively.

Neonates with low-grade GMH-IVH exhibited lower MK values in the PLIC compared to controls (0.541 ± 0.134 and 0.623 ± 0.189, respectively; P < 0.05 and FDR P < 0.1) and lower RK values (0.704 ± 0.178 and 0.930 ± 0.241, respectively; P < 0.001 and FDR P < 0.1). Additionally, these neonates demonstrated significantly lower MK (0.327 ± 0.073 and 0.431 ± 0.087, respectively; P < 0.05 and FDR P < 0.1) and RK (0.411 ± 0.135 and 0.574 ± 0.106, respectively; P < 0.001 and FDR P < 0.1) values in the thalamus. Similarly, neonates with low-grade GMH-IVH presented significantly lower RK than controls (0.384 ± 0.088 and 0.416 ± 0.131, respectively; P < 0.05 and FDR P < 0.1) in the caudate nucleus. Therefore, MK and RK in the PLIC, MK and RK in the thalamus and RK in the caudate nucleus exhibited significantly differences between the two groups. No regions of interest (ROIs) exhibited significantly lower FA values in low-grade GMH-IVH neonates compared to controls (P > 0.05 and FDR P > 0.1), and no differences in GCC were observed (P > 0.05 and FDR P > 0.1) (Table 2). Additionally, we selected the occipital lobe as the control ROI, and there were no differences between the two groups. The ICCs for DKI-derived metric measurements in each brain region were generally good or excellent (range, 0.756–0.985) (Supplementary Table 1).

**Table 2:** Measured Values in Brain Regions.

ROI	Parameter	Neonates with low-grade GMH-IVH (n = 30)	Preterm neonates (n = 40)	*P* Value	FDR *P* value
PLIC	FA	0.486 ± 0.121	0.495 ± 0.131	0.483	0.526
	MK	0.541 ± 0.134	0.623 ± 0.189	0.028*	0.093^†^
	RK	0.704 ± 0.178	0.930 ± 0.241	< 0.001***	0.012^†^
Thalamus	FA	0.181 ± 0.13	0.193 ± 0.22	0.493	0.537
	MK	0.327 ± 0.073	0.431 ± 0.087	0.031*	0.093^†^
	RK	0.411 ± 0.135	0.574 ± 0.106	0.045*	0.096^†^
GCC	FA	0.466 ± 0.04	0.479 ± 0.07	0.327	0.490
	MK	0.507 ± 0.544	0.519 ± 0.387	0.629	0.629
	RK	0.612 ± 0.373	0.644 ± 0.965	0.492	0.536
Caudate nucleus	FA	0.05 ± 0.03	0.06 ± 0.05	0.064	0.109
	MK	0.454 ± 0.072	0.436 ± 0.098	0.052	0.103
	RK	0.384 ± 0.088	0.416 ± 0.131	0.048*	0.096^†^

Unless otherwise indicated, results are represented as mean ± standard deviation for continuous variables. *Low-grade GMH-IVH* low-grade GMH-IVH = low-grade Germinal Matrix Hemorrhage-Intraventricular Hemorrhage, *FDR* false discovery rate, *ROI* region of interest, *PLIC* posterior limbs of the internal capsule, *GCC* genu of corpus callosum, *FA* Fractional anisotropy, *MK* mean kurtosis, *RK* radial kurtosis.

*P value < 0.05.

***P value < 0.001.

^†^FDR *P* Value < 0.1.

### Neurodevelopmental outcomes

Lower neurodevelopmental outcomes were observed in infants with low-grade GMH-IVH. Out of the 70 neonates subjected to neurodevelopmental assessment using Bayley-II scales of infant development at 18 months of age, significant differences emerged between neonates with low-grade GMH-IVH and controls across various parameters, as outlined in Table 3. Specifically, there were notable disparities in MDI scores (91.533 ± 8.775 and 120.344 ± 6.409 for low-grade GMH-IVH and controls, respectively; P < 0.001). Similarly, significant differences were noted in PDI scores between neonates with low-grade GMH-IVH and controls (91.533 ± 10.007 and 121.922 ± 7.912, respectively; P < 0.001).

**Table 3:** Neurodevelopmental scores at 18 months by using *Bayley*—II scales of infant development.

	Neonates with low-grade GMH-IVH (n = 30)	Preterm Neonates (n = 40)	*P* Value
MDI	91.533 ± 8.775	120.344 ± 6.409	< 0.001***
PDI	97.300 ± 10.007	121.922 ± 7.912	< 0.001***

*Low-grade GMH-IVH* low-grade GMH-IVH = low-grade Germinal Matrix Hemorrhage-Intraventricular Hemorrhage, *Bayley—II* Bayley Scales of Infant and Toddler Development 2nd Edition, *MDI* Mental Development Index, *PDI* Psychomotor Development Index.

***P value < 0.001.

### MRI metrics are associated with neurodevelopmental outcomes

**Figure 3:**
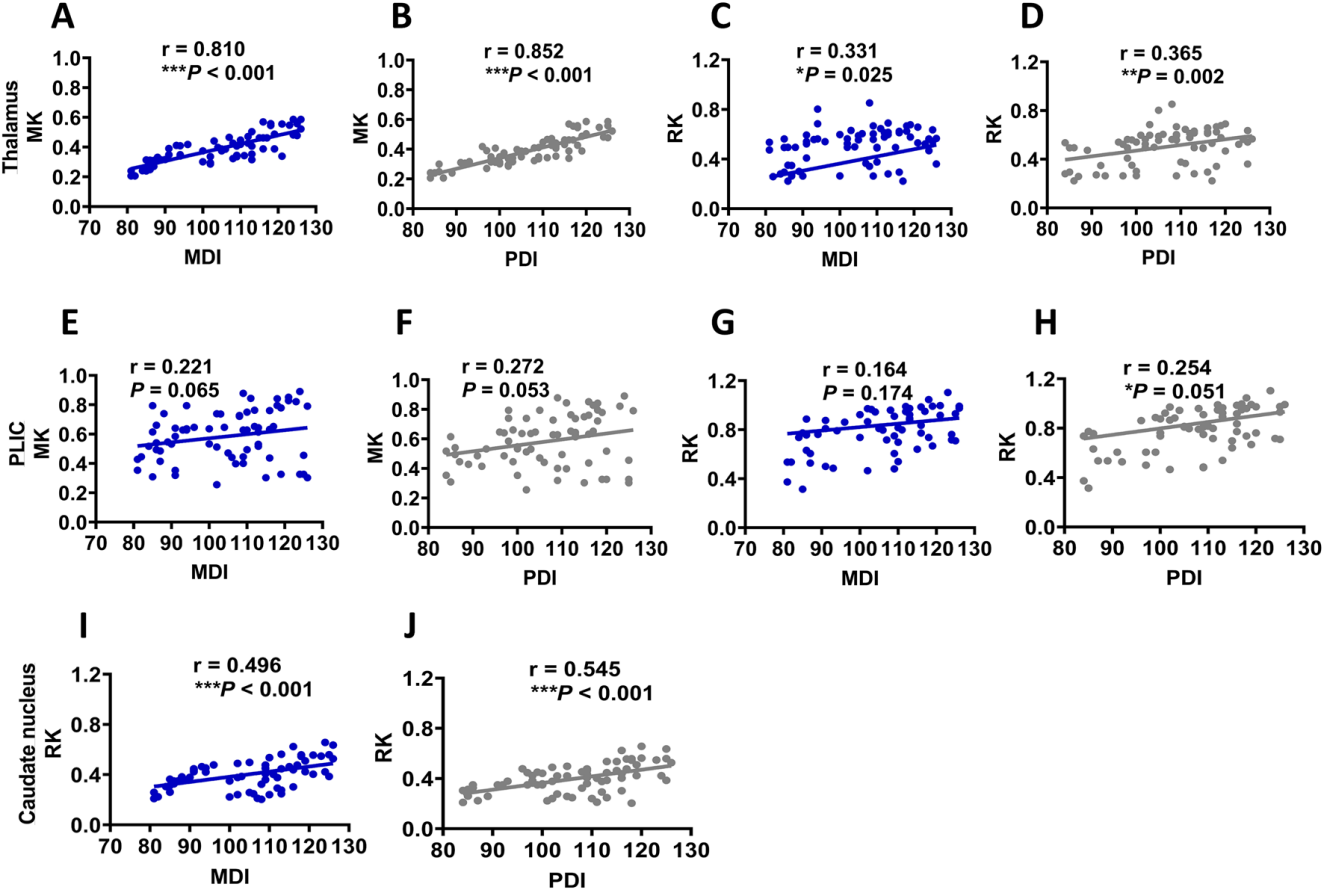
Correlation between DKI-derived metrics or volume and MDI and PDI scores at 18 months for neonates with low-grade GMH-IVH. (**A**–**D**) Pearson correlation between MK or RK in the thalamus and MDI or PDI scores. (**E**–**H**) The relationship between MK or RK in the PLIC and MDI or PDI scores. (**I**,**J**) The correlation between RK in the caudate nucleus and MDI or PDI scores. *DKI* Diffusion kurtosis imaging, *MDI* Mental Development Index, *PDI* Psychomotor Development Index, *MK* mean kurtosis, *RK* radial kurtosis, *low-grade GMH-IVH*  low-grade Germinal Matrix Hemorrhage-Intraventricular Hemorrhage, *PLIC* Posterior Limbs of the Internal Capsule.

Notably, the MK value in the thalamus of neonates with low-grade GMH-IVH exhibited a significant association with MDI (r = 0.810, 95% CI, 0.695–0.13; P < 0.001) and PDI (r = 0.852, 95% CI, 0.722–0.912; P < 0.001) scores (see Fig. 3A,B). Concurrently, the RK value in the caudate nucleus demonstrated a significant positive correlation with MDI (r = 0.496, 95% CI, 0.657–0.933; P < 0.001) and PDI (r = 0.545, 95% CI, 0.712–0.942; P < 0.001) scores for neonates with low-grade GMH-IVH (see Fig. 3I,J). Notably, no significant correlations were observed between other DKI-derived metrics and neurodevelopmental outcomes (see Fig. 3C–H).

### Diagnostic performance of DKI metrics in different brain regions for predicting adverse neurodevelopmental outcomes

The ROC curve and AUC were used to assess diagnostic performance of MK and RK in thalamus and caudate nucleus for predicting MDI and PDI. The differential diagnosis ability of MK and RK was found to be good in thalamus (AUC = 0.866 and 0.787) and caudate nucleus (AUC = 0.833 and 0.671) (see Table 4).

**Table 4:** Diagnostic performance of DKI metrics in different brain regions for predicting adverse neurodevelopmental outcomes.

		AUC	Sensitivity (%)	Specificity (%)	Accuracy (%)
MDI	MK -TH	0.866 (0.763–0.943)	70 (43.1, 95.2)	72.8 (64.1, 96.6)	78.2
	RK- Caudate nucleus	0.833 (0.782–0.823)	69.8 (45.5, 86.1)	90.11 (75.2, 96.3)	85.3
PDI	MK -TH	0.787 (0.624–0.946)	67.2 (54.6, 75.8)	89.5 (73.1, 96.9)	75.4
	RK—Caudate nucleus	0.671 (0.597–0.764)	58.9 (39.6, 89.6)	80.1 (70.4, 91.5)	69.6

*AUC* area under the curve, *TH* thalamus, *MK* mean kurtosis, *RK* radial kurtosis.

## Discussion

This case–control study reveals abnormal microstructural alterations in the brains of neonates with low-grade GMH-IVH, suggesting a potential link between these abnormalities and neurodevelopmental outcomes at 18 months within this subgroup.

Specifically, our findings highlight altered DKI metrics in early- myelinated supratentorial white matter regions, including the PLIC, GCC, thalamus, and caudate nucleus, in neonates with low-grade GMH-IVH compared to controls. We observed reduced MK and RK, with no significant differences in FA values. MK is generally proportional to the heterogeneity and complexity of brain microstructure. An increase in MK may indicate more densely packed cells or higher cellular complexity, while a decrease may suggest loss of cellular structure^21^. Low-grade GMH-IVH can trigger inflammation in adjacent in adjacent white matter through activated microglia, red blood cell passage, and red blood cell degradation^22^. Such conditions can impair both white and gray matter in this toxic environment^23^. Additionally, RK evaluation may serve as a novel method for assessing the integrity of cell membranes and surrounding myelin sheaths^7^. Intriguingly, simultaneous decreases in MK and RK have been observed in other regions of brain injury resulting from axonal loss, followed by a decrease in the extracellular matrix and glial cells^12,24,25^. On the other hand, the lack of significant changes in FA values suggests that MK and RK metrics can reflect subtle differences, likely indicating milder microstructural alterations, such as minimal fiber loss without gross tissue damage^7^. So MK and RK values can be more sensitive to detect damage to microstructures.

Our investigation identified significant deviations in MK within the thalamus and RK within the caudate nucleus, all of which emerged as pivotal factors associated with adverse outcomes in both MDI and PDI. This aligns seamlessly with existing literature that underscores the impact of low-grade GMH-IVH on macroscopic thalamus and caudate nucleus development, thereby contributing to compromised neurodevelopmental outcomes^12,26,27^. Supporting our findings, Han Y et al. highlighted a correlation between neurodevelopmental outcomes and RK values in the caudate nucleus among neonates with encephalopathy^21^. Additionally, Jeanie L et al.'s prior work demonstrated a connection between thalamus abnormalities and MDI / PDI scores at 18 months of age^28^. The noteworthy impact of low-grade GMH-IVH on thalamus and caudate nucleus development stems from the germinal matrix's extension along the lateral ventricles until approximately 28 weeks' gestational age, influencing the crucial development of these structures^29^. The thalamus, responsible for processing all sensory information (except smell)^30^, and the caudate nucleus, playing a critical role in various higher neurological functions^31^, are particularly susceptible to impairment when brain damaged. Our study revealed a clear association between alterations in white matter microstructure, and subsequent neurodevelopmental impairments at 18 months of age in a subset of the neonates under investigation. To better discern infants at risk of adverse neurological outcomes due to low-grade GMH-IVH, we conducted an ROC analysis to assess the diagnostic efficacy of DKI metrics across various ROIs. Our findings revealed that MK and RK exhibited strong predictive capabilities, particularly in the thalamus and caudate nucleus regions, for forecasting MDI and PDI outcomes. The maturation of the thalamus and caudate nucleus is closely linked to both cognitive and physical functionalities^32,33^. MK and RK are sensitive indicators for detecting the development level of nerve fiber tracts^34^. Therefore, we posit that the identification of abnormal fiber tract development in these specific ROIs by MK and RK serves as a predictive marker for alterations in both MDI and PDI parameters.

Several limitations characterize our study. The relatively small sample size, although justified by the low incidence rate of low-grade GMH-IVH, necessitates cautious interpretation of the findings. Additionally, the neurodevelopmental follow-up was relatively short, limited to a subset of neonates at 18 months of age during the study period. Another constraint is the reliance on a single system for data collection. Future investigations should aim to gather data from diverse systems and time points to enhance both volume and variety of information.

## Conclusions

Our study underscores the significant associations between MK in the thalamus, and RK in the caudate nucleus alterations with cognitive and motor outcome scores at 18 months. These findings emphasize the potential relevance of these specific metrics as early indicators of cognitive and motor development in neonates with low-grade GMH-IVH. Precise anticipation of outcomes based on early DKI metrics holds the potential to enable earlier interventions for infants at risk and could greatly enhance the efficacy of intervention strategies. We hope to leverage this information to enhance clinical outcomes for neonates affected by low-grade GMH-IVH.

### Supplementary Information

Supplementary Information.

## Data Availability

The datasets used and/or analyzed during the current study available from the corresponding author on reasonable request.

## Abbreviations

DKI: Diffusion kurtosis imaging
MK: Mean kurtosis
RK: Radial kurtosis
DTI: Diffusion tensor imaging
GMH-IVH: Germinal matrix hemorrhage-intraventricular hemorrhage
GA: Gestational age
MDI: Mental development index
PDI: Psychomotor development index
ROI: Region of interest
PLIC: Posterior limbs of the internal capsule
GCC: Genu of corpus callosum
