# Supplementary material for: Associations between diffusion kurtosis imaging metrics and neurodevelopmental outcomes in neonates with low-grade germinal matrix and intraventricular hemorrhage
Source: Sci Rep. 2024 Jul 16;14:16455. doi: 10.1038/s41598-024-67517-5 (PMC11252380; doi:10.1038/s41598-024-67517-5)
Supplement: Supplementary file 1 — Supplementary Information. [file 41598_2024_67517_MOESM1_ESM.docx]

**Supplementary**

**S1** **Diagnostic criteria**

The severity of hemorrhage on ultrasonography is graded based upon the location and extent of the GMH-IVH and presence of lateral ventricular dilatation:

Grade I – Either:

•Bleeding is confined to the germinal matrix (ie, GMH only), or

•GMH plus IVH occupying <10 percent of the lateral ventricular area

Grade II – IVH that occupies 10 to 50 percent of the lateral ventricle area.

Grade III – IVH that occupies >50 percent of the lateral ventricle area and is associated with acute ventricular dilatation.

Periventricular hemorrhagic infarction (PVHI; previously referred to as Grade IV IVH) – Hemorrhagic infarction in periventricular white matter ipsilateral to large IVH.

"Low-grade" GMH-IVH refers to grades I and II and "severe" refers to grades III and PVHI (grade IV).

**S2 Bayley - II scales of infant development (BSID)**

The test included three parts: intelligence scale, motor scale, and behavior record. Results: The results of mental development index (MDI) and psychomotor development index (PDI) were scored according to the distribution of developmental index levels. A score of ≥130 is very excellent, 120 to 129 is excellent, 110 to 119 is above medium, 90 to 109 is medium, 80 to 89 is below medium, 70 to 79 is critical, and ≤69 is stunted. Normal mean = 100, standard deviation = 16.

**Table S1 MRI Acquisition Protocol for preterm neonate**

| **Parameter** | **T1WI** | **T2WI** | **DKI** | **Synthetic MRI** |
| --- | --- | --- | --- | --- |
| Field of view (mm) | 200 x 200 | 200 x 200 | 256 x 256 | 512 x 512 |
| Matrix | 320 x 320 | 320 x 320 | 96 x 96 | 288 x 224 |
| Repetition time (msec) | 3010 | 4500 | 2000 | 4000 |
| Echo time (msec) | 14 | 99 | 103.3 | 19.6 |
| Inversion time (mse) | - | - | - | 28.1 |
| Number of averages | 1 | 1 | 1 | 1 |
| Flip angle (degrees) | 12 | 12 | 90 | 90 |
| Section thickness/gap | 4/0 | 4/0 | 4/0 | 3/0 |
| Number of directions | NA | NA | 30 | NA |
| Acquisition time | 2 min | 2 min | 7 min | 5 min |

**Table S2 Inter-observer consistency of measurements**

| **Parameters** | **Intraclass correlation coefficient, 95% CI** |
| --- | --- |
| FA | 0.955(0.866 - 0.985) |
| MK | 0.943 (0.756 - 0.982) |
| RK | 0.914 (0.756 - 0.973) |

95% CI = 95% confidence interval.

FA = Fractional anisotropy, MK = mean kurtosis, RK = radial kurtosis.
